# UCP1 alleviates renal interstitial fibrosis progression through oxidative stress pathway mediated by SIRT3 protein stability

**DOI:** 10.1186/s12967-023-04376-0

**Published:** 2023-08-02

**Authors:** Wei Xiong, Zhiyong Xiong, Anni Song, Chuntao Lei, Chen Ye, Hua Su, Chun Zhang

**Affiliations:** 1 Department of Nephrology, Union Hospital, Tongji Medical College, Huazhong University of Science and Technology, Wuhan, 430022 China; 2 Department of Urology, Union Hospital, Tongji Medical College, Huazhong University of Science and Technology, Wuhan, 430022 China

**Keywords:** Renal interstitial fibrosis, UCP1, Reactive oxygen species (ROS), SIRT3, Chronic kidney disease (CKD)

## Abstract

**Background:**

Renal interstitial fibrosis is a common pathway for the progressive development of chronic renal diseases (CKD) with different etiology, and is the main pathological basis leading to end-stage renal disease. Although the current research on renal interstitial fibrosis is gradually deepening, the diagnosis and treatment methods are still very lacking. Uncoupling protein 1 (UCP1) is a nuclear encoded protein in mitochondria inner membrane and plays an important role in regulating energy metabolism and mitochondrial homeostasis. However, the biological significance of UCP1 and potential regulatory mechanisms in the development of CKD remain unclear.

**Methods:**

Unilateral ureteral obstruction (UUO) model was used to construct the animal model of renal fibrosis, and TGF-β1 stimulation of HK2 cells was used to construct the vitro model of renal fibrosis. UCP1 expression was detected by Western blot, immunoblot analysis and immunohistochemistry. UCP1 was upregulated by UCP1 overexpressing lentivirus and UCP1 agonist CL316243. Western blot and immunofluorescence were used to detect epithelial mesenchymal transition (EMT)-related markers, such as collagen I, fibronectin, antioxidant enzyme SOD2 and CAT. Reactive oxygen species (ROS) production was detected by ROS detection kit. SIRT3 knockdown was performed by siRNA.

**Results:**

This study presents that UCP1 is significantly downregulated in patients with renal fibrosis and UUO model. Further studies discover that UCP1 overexpression and CL316243 treatments (UCP1 agonists) reversed EMT and extracellular matrix (ECM) accumulation in renal fibrosis models in vivo and in vitro. Simultaneously, UCP1 reduced the ROS production by increasing the stability of SIRT3. When SIRT3 was knocked down, the production of ROS decreased.

**Conclusions:**

Elevating the expression of UCP1 can inhibit the occurrence of oxidative stress by stabilizing SIRT3, thereby reducing EMT and ECM accumulation, and ultimately alleviating renal interstitial fibrosis. It will provide new instructions and targets for the treatment of CKD.

**Supplementary Information:**

The online version contains supplementary material available at 10.1186/s12967-023-04376-0.

## Background

Renal interstitial fibrosis, manifested by the accumulation of scarring within the renal parenchyma, is the common ultimate pathway for almost all chronic and progressive renal diseases [1]. Chronic kidney disease (CKD) is a chronic nephrotic syndrome caused by multiple factors with abnormal structure and function of the kidney. When the disease develops to a serious condition, dialysis or kidney transplantation are often needed to perform renal replacement therapy to prolong the patient's life [2]. According to statistics, the global incidence of CKD is about 10% [3]. Therefore, delaying the progression of renal interstitial fibrosis can not only prolong the time from drug therapy to renal replacement therapy, but also reduce the economic pressure on patients' families and save medical resources. However, there is currently no specific drug for renal interstitial fibrosis, which cannot reverse its progression to end-stage renal disease (ESRD).

Renal interstitial fibrosis is an irreversible organic change in the kidney caused by a variety of pathogenic factors characterized by epithelial mesenchymal transformation (EMT), excessive accumulation of inflammatory cells and extracellular matrix, activated myofibroblasts and renal ischemia and hypoxia [4]. They are the basic pathological processes for all CKD to eventually leading to chronic renal failure. Tubular EMT is a process dominated by many factors. It completes the migration of tubular epithelial cells into the renal interstitium and differentiation into myoblasts in a complex way [5]. In the process of EMT, renal tubular epithelial cells lose their marker proteins and eventually become myofibroblasts with the ability to over secrete extracellular matrix (ECM) [5].

The main components of ECM are protein tissues such as collagen, elastin, and glycoproteins. Among them, collagen is processed by fibroblasts to become collagen fibers, which serve as an important part to provide support and connection for the kidneys under physiological conditions [6]. ECM is continuously generated in the kidney and degraded with metabolism, always maintaining a dynamic balance. In pathological conditions, the increased synthesis or decreased degradation of ECM breaks this balance, so that the normal kidney structure is replaced by a proliferating ECM [7]. At present, the regulatory mechanisms of EMT and ECM are not fully understood. For example, myofibroblast is the main source of ECM in renal fibrosis, but its cell origin has been controversial. Recent study using single-cell transcriptome sequencing to trace the genetic fate of mice, combined with histological analysis of human tissues, showed that epithelial cells, endothelial cells, hematopoietic cells, and mesenchymal cells all contribute to renal fibrosis [8].

Uncoupling protein 1 (UCP1) generally exists in brown fat and is closely related to tissue adaptive thermogenesis [9]. UCP1 dissipates the proton electrochemical gradient by catalyzing proton leakage into the mitochondrial inner membrane, so the energy produced by the oxidation of the respiratory matrix does not drive ATP synthase to produce ATP, but is released as heat. Therefore, the two processes of substrate oxidation and ADP phosphorylation are ‘uncoupled’ [10]. In mice, brown fat (BAT) thermogenesis has been shown to eliminate glucose and lipids in the blood and reduced the occurrence of metabolic diseases, while brown fat deficiency or UCP1 specific gene knockout induced obesity [11, 12]. Previous study by our research has also demonstrated that UCP1 reduced kidney damage by eliminating lipid accumulation in acute kidney injury [13].

However, in addition to being closely related to metabolism, UCP1 can also remove oxygen free radicals produced during oxidative respiration and decrease the level of intracellular oxidative stress through uncoupling [14]. Study has shown that UCP1 alleviated ischemia-reperfusion-induced AKI by inhibiting oxidative stress [15]. Oxidative stress is a also key pathological process in the development of renal fibrosis, which is manifested by ROS accumulation and decreased expression of antioxidant enzymes [16]. It can be seen that UCP1 may play a significant role in CKD, but the current research has not been reported.

The present study found that the expression of UCP1 was significantly decreased in patients, animals and cell models of renal interstitial fibrosis, systematically analyzed its function in renal interstitial fibrosis, and clarified its regulatory mechanism.

## Methods

### Animals

All animal experiments in this study were approved by the Ethics Committee of Huazhong University of Science and Technology, and carried out in conformity with the Guidelines for the Use and Care of Laboratory Animals of the National Institutes of Health and. Male C57BL/6 mice weighing 20–25 g and aged 6–8 weeks were used, provided by Charles River (Beijing, China). All mice were raised using standard methods. During the operation, all animals were anesthetized by intraperitoneal injection of pentobarbital sodium (50 mg/kg), and the left ureter was completely blocked with 4–0 nylon to establish UUO model. Sham operated mice were used as control. The kidney was taken 3, 7, 14 days after the operation. The 7-day UUO model was selected as the model for the follow-up intervention experiment. CL316243 (Tocris Bioscience, Bristol, UK) was injected continuously through the caudal vein and intra peritoneum for 7 days at a dose of 2 mg kg^−1^ d^−1^. The control group was injected with normal saline in the same way. The mice were placed in a metabolic cage to collect urine the day before execution. The total amount was recorded and the 24 h urine protein was detected by biuret method. The supernatant of rat urine was collected, and the content of rat urine albumin was detected by enzyme-linked immunosorbent assay (ELISA) according to the instructions of ELISA kit. Creatinine oxidase method was used to determine urinary creatinine content following the instructions of the creatinine assay kit. The ratio of urinary albumin to creatinine is calculated as creatinine ratio level. Urine KIM-1 concentration was determined using ELISA according to the kit steps. Each experimental group and control group contained 5 mice.

### Intrarenal adenovirus delivery

Both the adenovirus overexpressing UCP1 and the control adenovirus were from Vigenebio (Shandong, China). Simultaneously with the establishment of the UUO model, the virus was injected into the mice kidney by intrarenal injection. Mice were transiently anesthetized before injection of the virus, and the abdominal cavity was surgically opened to expose one side of the kidney. Then 100 μL UCP1 overexpressed adenovirus or control adenovirus (1 × 10^11^ pfu mL^−1^) was absorbed with 31G needle. From three to five sites in a single kidney, the virus was injected into the renal cortex at a slow pace.

### Human renal biopsy samples

The patient samples were taken from the Department of Nephrology, Wuhan Union Medical College Hospital, and the kidney biopsy samples were obtained from the clinical diagnostic procedure. Control samples were obtained from the kidney poles of individuals who underwent tumor nephrectomy and did not have any other kidney diseases. Both healthy and patients with TIF specimens contain 3 cases. The procedure of human kidney biopsy was approved by the Ethics Committee of Tongji Medical College, Huazhong University of Science and Technology. The kidney specimens were fixed with 10% formalin and paraffin embedded. The 3 μm thickness of the incision was used for immunohistochemical staining.

### Immunohistochemical and immunofluorescent staining

For immunohistochemical staining, kidney tissue was fixed with formalin and embedded in paraffin. The embedded tissue was cut into 4-μm slices for immunohistochemical staining. After the sections were dewaxed and rehydrated, to facilitate antigen extraction, they were incubated with EDTA for 5 min at 120 °C, followed by 3% H_2_O_2_ for 15 min at room temperature, and finally blocked with fetal bovine serum. Anti-UCP1 (ab10983, 1:100; Abcam, Cambridge, UK) or anti-SIRT3 (A7307, 1:100; ABclonal, Woburn, MA, USA) antibodies with slices were incubated at 4 °C overnight. The slices were then washed in phosphate buffered brine and incubated with biotinylated goat anti-rabbit antibodies (Beyotime, Jiangsu, China) for 20 min. The newly prepared diaminobenzidine was added to the slices and incubated at room temperature for 2–10 min. Finally, the sections were stained with hematoxylin, dehydrated with alcohol gradient, sealed, and examined under an optical microscope.

For immunofluorescent staining, fresh tissues were immersed in 4% paraformaldehyde for 6–8 h, and then transferred to 20% sucrose to allow the tissues to sink to the bottom and form frozen tissue blocks. Frozen tissues were sliced with a cryostat, and preserved at − 20 °C after 30 min at room temperature. When performing immunofluorescence experiments, the frozen sections were taken out of the refrigerator and left at room temperature for 30 min. The sections were then rinsed with PBS and sealed with 2% BSA at room temperature for 1 h. The primary antibody (Fibronectin, 15613-1-AP, 1:250; Proteintech, Manchester, UK) incubated overnight at 4 °C. Alexa Fluor 488-conjugated donkey anti-rabbit IgG (H + L) (AS035, 1:250; ABclonal, Woburn, MA, USA) was incubated as secondary antibodies. Finally, nuclei were stained with 4, 6-diaminyl-2-phenylindole (DAPI). The apoptotic cells were evaluated by the situ Apoptosis Detection kit (Roche, Mannheim, Germany).

### Hematoxylin–eosin (HE) and Masson staining

For HE staining, the slices were firstly put in xylene I and xylene II for 10 min, then placed in alcohol of different concentrations for dehydration, and finally washed with distilled water. Sections were incubated and stained with Harris hematoxylin for 3–8 min. Sections were rinsed and differentiated by adding 1% hydrochloric alcohol for a few seconds, returned to blue with 0.6% ammonia, and then rinsed with running water. Sections were stained with eosin solution for 1 to 3 min, and then dehydrated in different concentrations of ethanol and xylene. Finally, the slices were taken out to dry slightly and sealed with neutral glue. Examination, image acquisition and analysis were performed with a microscope.

The Masson staining procedure was firstly the same as HE staining. The paraffin sections were first dehydrated and stained with hematoxylin. The sections were then stained with Ponceau acid fuchsin solution for 5–10 min and quickly rinsed with distilled water. Sections were treated with aqueous phosphomolybdic acid aqueous solution for about 3–5 min and counterstained with aniline blue solution for 5 min. Differentiation was performed by 1% glacial acetic acid treatment for 1 min. Finally, dehydrate and mount the slides, observe and collect images under a microscope.

### Cell culture, treatment and transfection

Cells were cultured in an incubator at 37 °C and 5% CO_2_. The medium was MEM supplemented with 10% fetal bovine serum and 1% penicillin-streptomycin. The HK2 cell line was obtained from the American Type Culture Collection (Manassas, VA, USA). The cells were subcultured at a cell density of 80%, with fresh culture medium replaced 2–3 times a week, and subcultured at a ratio of 1:2–1:4. Cell propagation is limited to 15 times or less. Cells in logarithmic growth stage and in good growth state were selected and inoculated into 6-well plates at a cell density of 3 × 10^7^ cells /L after trypsin digestion, and 3 parallel multiwells were set in each group. Cells were treated with TGF-β1 (10 ng/μl; Proteintech) for 12–48 h, with 48 h being chosen as the stimulation time for follow-up experiments. CL316243 (Tocris Bioscience) was treated simultaneously to cells together with TGF-β1 at 1 μM concentration for 48 h before cells were collected. Cycloheximide (CHX) was bought from MCE and was added at 10 μg/mL at the indicated time points. The lentivirus overexpressing UCP1, SIRT3 and the corresponding control vector were bought from Genechem (Shanghai, China) and transfected according to the instruction manual. SIRT3 small interfering RNA (siRNA) was obtained from Ribobio (Guangzhou, China). MDA content determination kit was provided by Nanjing Jiancheng and operated according to the steps in the instruction. Oil red O was obtained from Wuhan Servicebio technology (Wuhan, China).

### Western blotting assays

The collected cells and tissues were lysed with RIPA lysis buffer (Beyotime) for protein extraction. Protein samples were calculated and prepared by BCA protein quantification method. Firstly, the working solution was calculated and prepared according to the instructions of the BCA protein quantitative kit, and the standard product and the sample to be tested were added. BCA working solution was added and incubated for 30 min. Light absorption values of each sample were detected at A562nm. The concentration of the sample was calculated according to the standard curve. Next, 50 μg of the prepared protein samples was separated by sodium dodecyl sulfate-polyacrylamide gel electrophoresis and then transferred to a polyvinylidene fluoride membrane (Millipore Corp., Bedford, MA, USA) for transmembrane. Membranes were enclosed in 5% nonfat dry milk (Beyotime) at room temperature for 1 h and incubated with primary antibodies overnight at 4 °C. Then, the membrane was immersed in blocking buffer containing secondary antibody (1:2500; AntGene, Wuhan, China) for 2 h before detection. Glyceraldehyde-3-phosphate dehydrogenase (GAPDH) served as an internal parameter. Primary antibodies were E-cadherin (20874-1-AP, 1:1000; Proteintech), α-SMA (14395-1-AP, 1:1000; Proteintech), Fibronectin (15613-1-AP, 1:1000; Proteintech), Collagen I (14695-1-AP, 1:1000; Proteintech), SOD2 (A1340, 1:1000; ABclonal), CAT (A11777, 1:1000; ABclonal), SIRT3 (A7307, 1:1000; ABclonal), UCP1 (ab10983, 1:1000; Abcam), GAPDH (60004-1-Ig, 1:1000; Proteintech), LC3 (14600, 1:1000; Proteintech, Manchester, UK). Density analysis of western blot images was performed by ImageJ2x. SPSS Statistics 22.0 (IBM SPSS, Chicago, IL) and Excel 2016 (Microsoft) were used for statistical analysis.

### RNA isolation and real-time PCR analysis

RNA was extracted from tissues using TRIzol reagents (Takara, Dalian, China) and then reverse-transcribed into cDNA using PrimeScriptRT Master Mix (Takara) according to the manufacturer's instructions. MRNA levels of target genes were measured using a NanoDrop 2000 spectrophotometer (NanoDrop Technologies, Wilmington, DE, USA). SYBR Green mix (thermoscientific, Waltham, MA, USA) was used for quantitative PCR analysis. Samples were normalized by GAPDH. The primer sequence is as follows (Table 1):

**Table 1:** List of primers for PCR of mice

Primer	Sequence
GAPDH	
Forward	5′-ATGGTGAAGGTCGGTGTGAA-3′
Reverse	5′-TGGAAGATGGTGATGGGCTT-3′
E-cadherin	
Forward	5′- TCGGAAGACTCCCGATTCAAA-3′
Reverse	5′- CGGACGAGGAAACTGGTCTC-3′
α-SMA	
Forward	5′- GGCACCACTGAACCCTAAGG-3′
Reverse	5′- ACAATACCAGTTGTACGTCCAGA-3′
SOD2	
Forward	5′- AGACCTGCCTTACGACTATGG-3′
Reverse	5′- CTCGGTGGCGTTGAGATTGTT-3′
CAT	
Forward	5′- TGGCACACTTTGACAGAGAGC-3′
Reverse	5′- CCTTTGCCTTGGAGTATCTGG-3′

### Oil red staining

Cells were cultured in 6-well plates until 30% fused. The oil red dye was prepared with the ratio of saturated oil red to ultra-pure water 2:3. Cell medium was removed and cells were washed twice with PBS. The cells were fixed with 4% paraformaldehyde, then removed, and the sample was allowed to dry naturally after 10 min. The prepared oil red fuel was added, stained at room temperature for 30 min, and then the excess oil red dye was removed with PBS, dried and observed under microscope.

### Cellular reactive oxygen species (ROS) detection

Cellular ROS detection assay kit (deep red fluorescence) was bought from Abcam (ab186029; deep red fluorescence). The experimental process strictly followed the product instructions. First, cells were cultured in an appropriate medium and treated in a specific manner to induce ROS production. The cells were then stained with ROS deep red dye working solution and incubation at 37 °C for 30–60 min. Finally, the change of fluorescence intensity was monitored under fluorescence microscope (Ex/m = 650/675 nm).

### Statistical analysis

All data were presented as mean ± SEM. T-test was applied for two groups comparation and one-way analysis of variance (ANOVA) was applied for more than two groups comparation. P < 0.05 was considered as the significant value.

## Results

### UCP1 is downregulated in the renal tubules in patients with TIF, UUO mice and TGF-β1 stimulated TECs

**Fig. 1:**
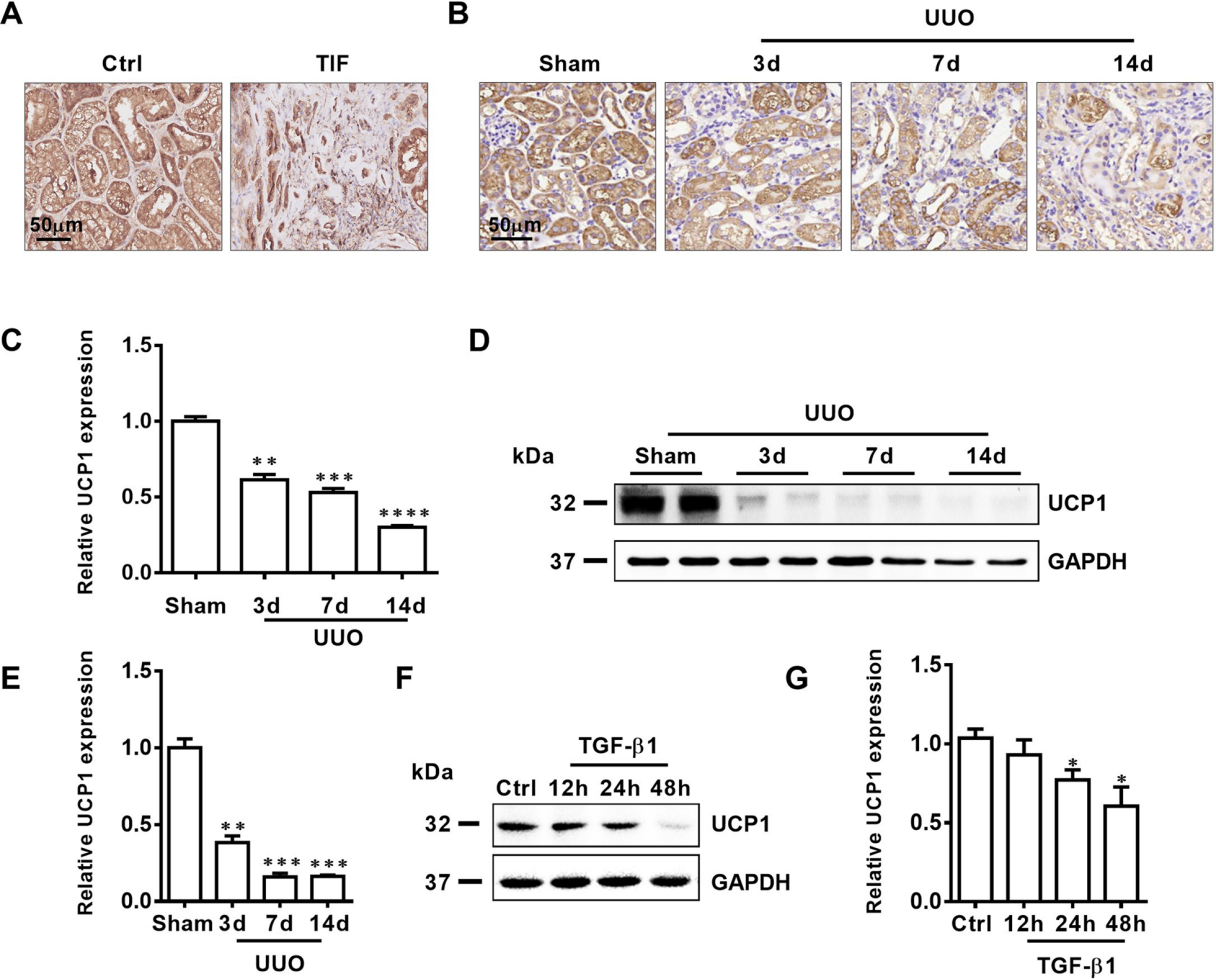
UCP1 is downregulated in TIF patients, UUO mice and TGF-β1 stimulated TECs. **A** Immunohistochemical images of UCP1 in the kidney cortex from patients with TIF and samples from nephrectomized patients. **B** Micrograph displaying immunohistochemical staining against UCP1 in UUO mice for 3, 7, and 14 days. **C** Aggregated statistics of integrated optical density (IOD)/area (n = 5). **D** Representative images and **E** corresponding quantification of UCP1 in UUO mice for 3, 7, and 14 days (n = 5). **F** Representative images and **G** corresponding quantification of UCP1 in HK2 cells exposed to TGF-β1 for 12, 24 and 48 h. * p < 0.05, ** p < 0.01, *** p < 0.001, **** p < 0.0001 vs. Ctrl or Sham

Our previous study confirmed that UCP1 was mainly expressed in the tubules of kidney, and was less presented in the glomerulus, medulla, and renal papilla [13]. By immunohistochemical staining, we found that the expression of UCP1 in the renal tubules of patients with renal interstitial fibrosis was significantly reduced (Fig. 1A). Subsequently, the animal model of renal interstitial fibrosis was set up by UUO (Additional file 1: Fig. S1). The results of immunohistochemistry and western analysis also confirmed that the expression of UCP1 in UUO model was significantly lower than that in normal mouse kidney tissue (Fig. 1B–E). In vitro, HK2 cells were stimulated with TGF-β1 to establish the cell model. Consistent with the previous results, UCP1 expression in HK2 cells was gradually reduced with the extension of TGF-β1 stimulation time (Fig. 1F, G). The above findings suggested that UCP1 might take part in the occurrence and development of renal interstitial fibrosis.

### Upregulation of UCP1 significantly alleviates EMT, ECM accumulation and renal interstitial fibrosis in UUO mice

**Fig. 2:**
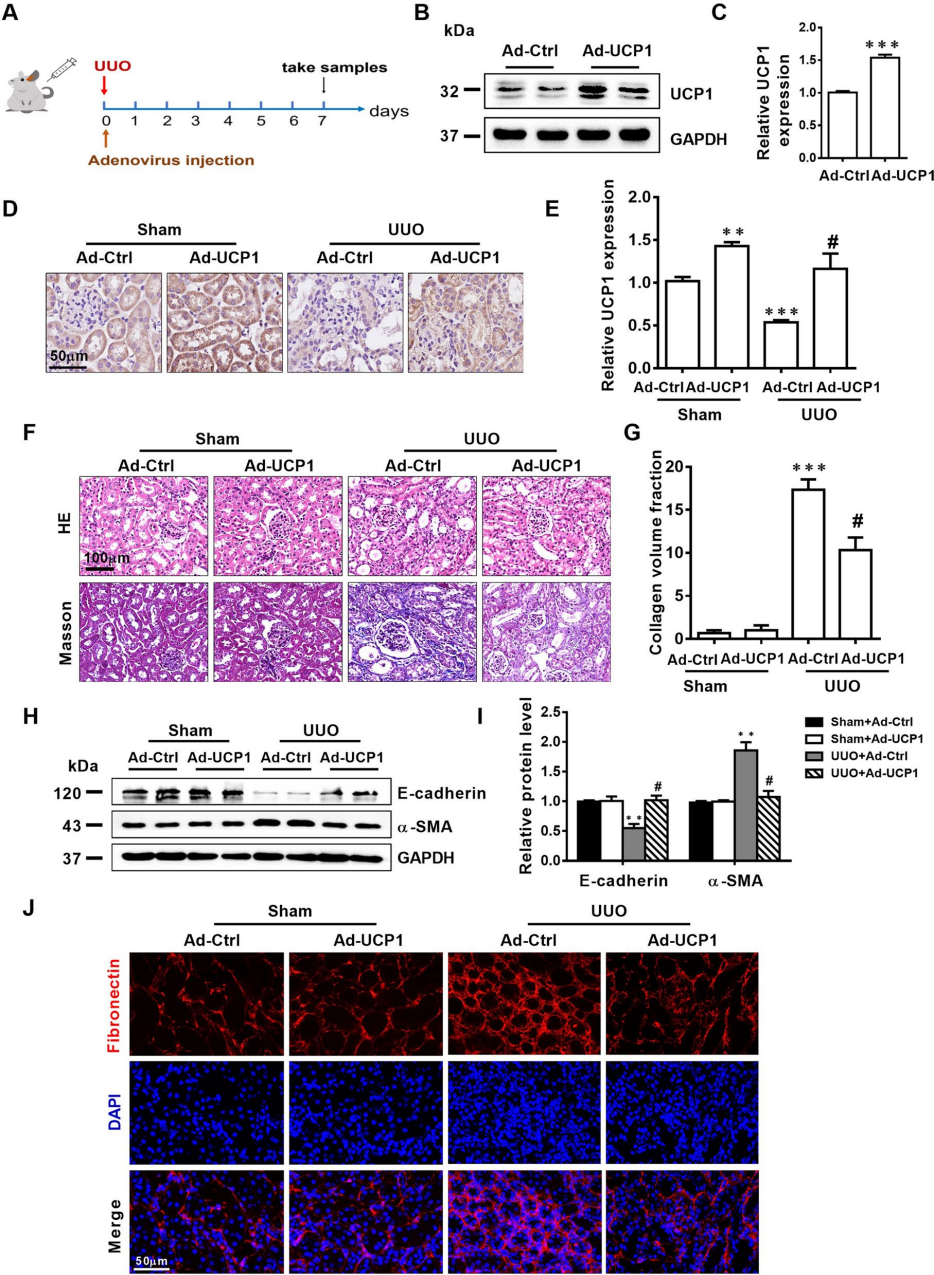
Upregulation of UCP1 alleviates EMT, ECM accumulation and renal interstitial fibrosis in UUO mice. **A** Specific administration methods of animal models. **B** Representative images and **C** corresponding quantification of UCP1 in animal models with UCP1-expressing adenovirus or blank control injection (n = 5). **D** Photomicrographs showing immunohistochemical staining against UCP1 in Sham and UUO mice with UCP1-expressing adenovirus or blank control injection. **E** Aggregated statistics of integrated optical density (IOD)/area (n = 5). **F** Representative photomicrographs of HE staining and Masson’s trichrome staining of sham and UUO animal models defined in D. **G** Relative collagen volume fraction of animal models defined in D (n = 5). **H** Representative images and **I** corresponding quantification of E-cadherin and α-SMA in animal models defined in D (n = 5). **J** Immunofluorescence images of Fibronectin in animal models defined in D. * p < 0.05, ** p < 0.01, *** p < 0.001, **** p < 0.0001 vs. Sham; # vs. UUO

Firstly, UCP1 overexpressed adenovirus was injected into renal parenchyma to construct an animal model with upregulated UCP1 expression. The specific administration method is shown in Fig. 2A. As shown in Fig. 2B–E, this manipulation significantly up-regulated UCP1 expression in the renal tubules of C57 mice. Hematoxylin (HE) and Masson staining revealed that renal morphology was greatly improved after overexpression of UCP1 in UUO model (Fig. 2F). Collagen volume fraction analysis showed that the level of renal interstitial fibrosis was significantly increased in UUO model, while overexpression of UCP1 could alleviate renal interstitial fibrosis (Fig. 2G). EMT and ECM accumulation are key pathophysiological process in renal interstitial fibrosis. Through western assay analysis and RT-PCR, the overexpression of mesenchymal cell marker α-SMA and the deletion of epithelial marker E-cadherin in UUO mice model were significantly improved after increasing the expression of UCP1 (Fig. 2H, I, Additional file 2: Fig. S2A, B). At the same time, the fibrosis marker Fibronectin was detected by fluorescence, and the results indicated that the expression of Fibronectin in the UUO mice model was reduced after overexpression of UCP1 (Fig. 2J).

### CL316243 treatment significantly alleviates EMT, ECM accumulation and renal interstitial fibrosis in UUO mice

**Fig. 3:**
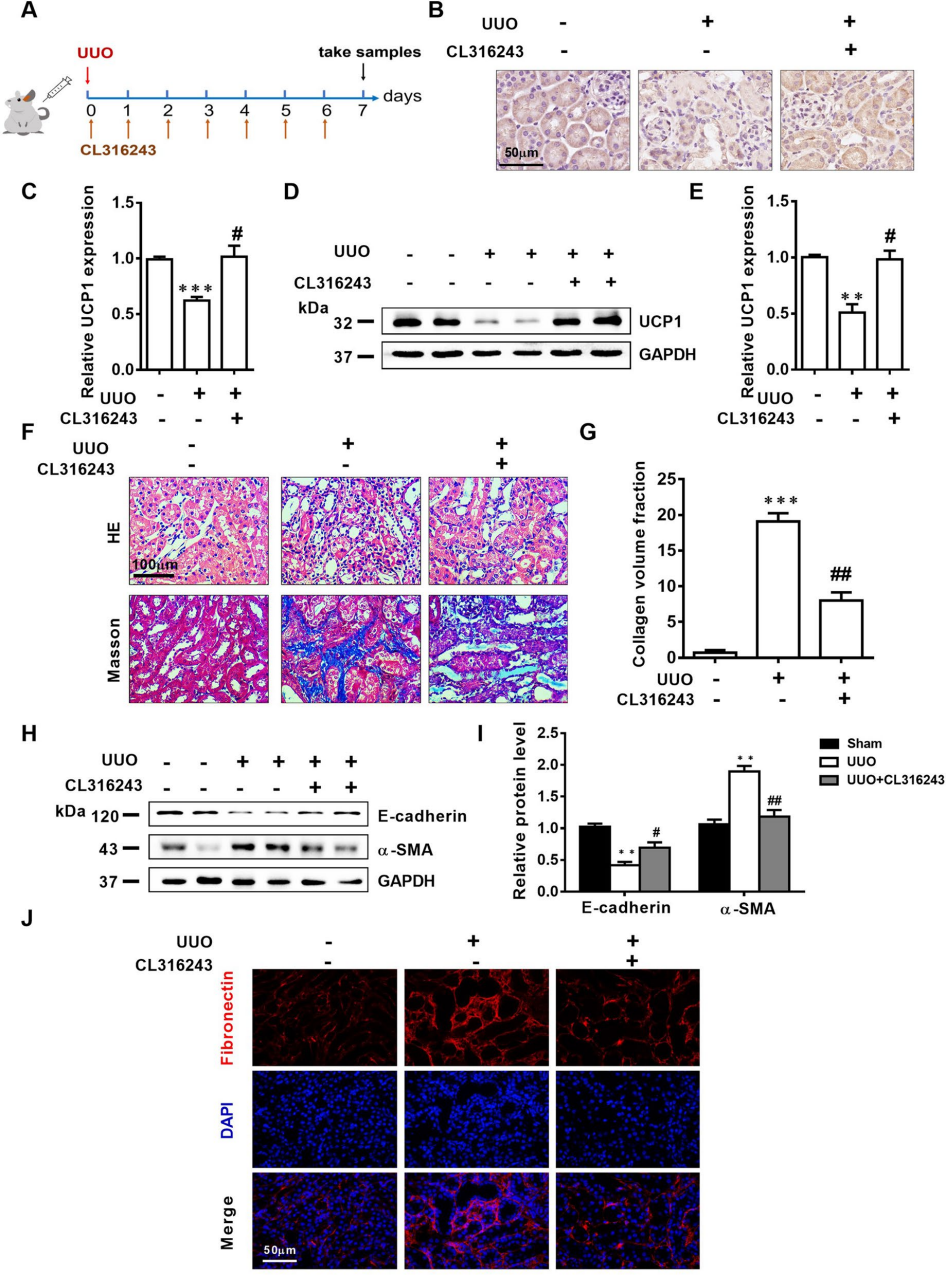
CL316243 treatment significantly alleviates EMT, ECM accumulation and renal interstitial fibrosis in UUO mice. **A** Specific administration methods of animal models. **B** Photomicrographs showing immunohistochemical staining against UCP1 in Sham and UUO mice with or without CL316243 treatment. **C** Aggregated statistics of integrated optical density (IOD)/area (n = 5). **D** Representative images and **E** corresponding quantification of UCP1 in animal models specified in B (n = 5). **F** Representative micrographs of HE and Masson staining of animal models defined in B. **G** Relative collagen volume fraction of animal models specified in B (n = 5). **H** Representative images and **I** corresponding quantification of E-cadherin and α-SMA in animal models defined in B (n = 5). **J** Immunofluorescence images of Fibronectin in animal models defined in B. * p < 0.05, ** p < 0.01, *** p < 0.001, **** p < 0.0001 vs. Sham; # vs. UUO

CL316243 is a potent and selective β3-adrenoceptor agonist that has been identified to elevate thermogenesis and metabolic rate in brown adipose tissue by specifically activating UCP1 [17]. Through continuous tail intravenous injection and intraperitoneal injection of CL316243 into UUO mice model (specific administration mode was shown in Fig. 3A), the results of immunohistochemistry staining and western blot assay analysis presented that the UCP1 expression in renal tubules was significantly upregulated (Fig. 3B–E). Further staining by HE and Masson indicated that the treatment of CL316243 obviously improved the morphology of kidney tissue, and the collagen volume fraction was also decreased compared with the UUO model group (Fig. 3F, G). Consistent with adenovirus injection, CL316243 treatment also notably reduced EMT levels and extracellular matrix deposition in the UUO model (Fig. 3H–J).

### Both upregulation of UCP1 and CL316243 treatment inhibit EMT and ECM accumulation in vitro

**Fig. 4:**
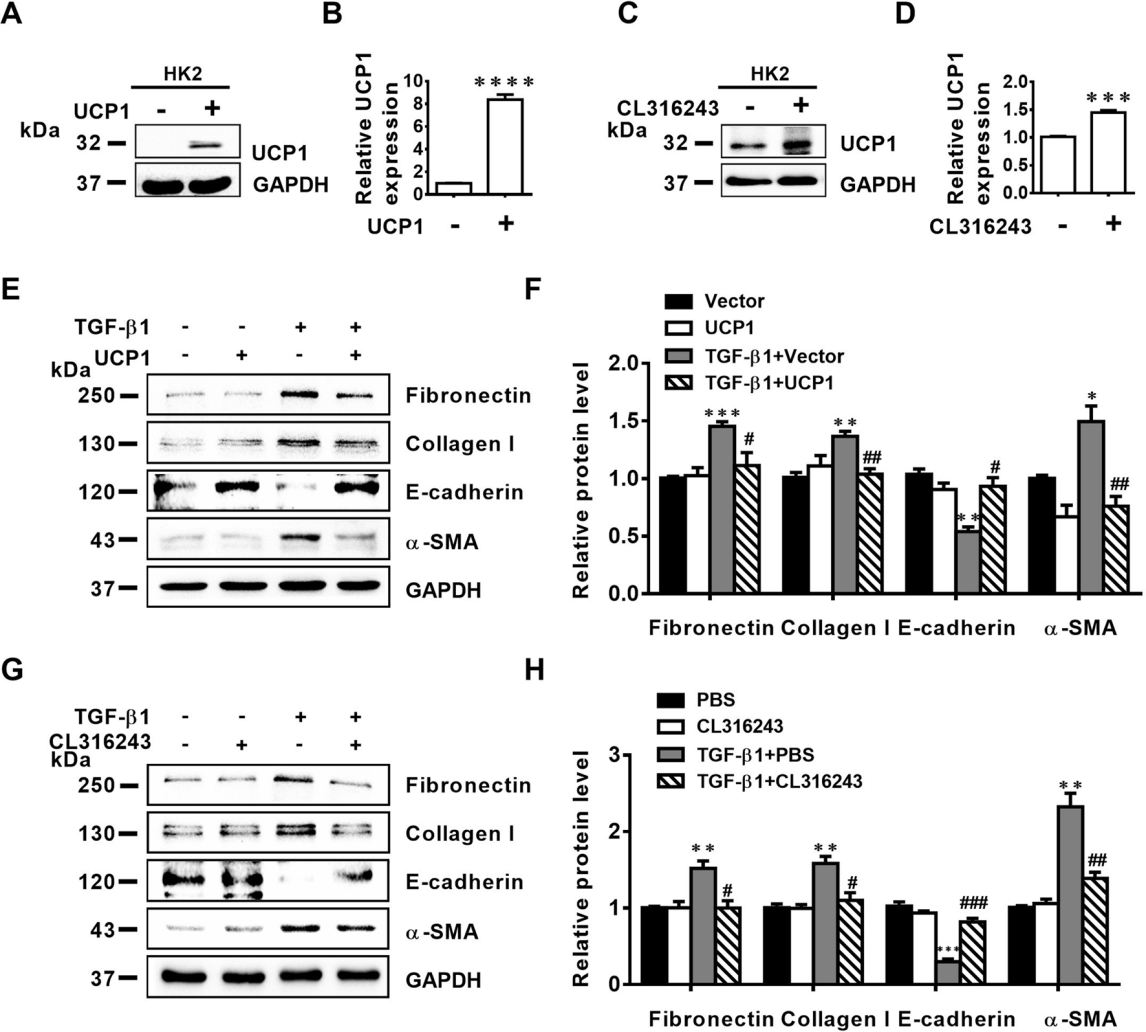
Upregulation of UCP1 and CL316243 treatment inhibits EMT and ECM accumulation in vitro. **A** Representative images and **B** corresponding quantification of UCP1 in HK2 cells in different cell groups. **C** Representative images and **D** corresponding quantification of UCP1 in HK2 cells in different cell groups. **E** Representative images and **F** corresponding quantification of Fibronectin, Collagen **I**, E-cadherin and α-SMA in HK2 cells in different cell groups. **G** Representative images and **H** corresponding quantification of Fibronectin, Molecular Collegen I, E-cadherin and α-SMA in HK2 cells in different cell groups. * p < 0.05, ** p < 0.01, *** p < 0.001, **** p < 0.0001 vs. Ctrl; # vs. TGF-β1

The previous study results have confirmed the important role of UCP1 in renal interstitial fibrosis in vivo, and it is essential to verify whether the same role is present at the cellular level. Firstly, we constructed HK2 cells overexpressing UCP1 by lentivirus transfection (Fig. 4A, B). Meanwhile, the expression of UCP1 was up-regulated by CL316243 stimulation (Fig. 4C, D). Furthermore, we constructed a cell model corresponding to renal interstitial fibrosis by stimulating HK2 cells with TGF-β1. As shown in Fig. 4E, F, the EMT-related marker α-SMA was increased along with the decreased E-cadherin expression under TGF-β1 stimulation, and this abnormal change was significantly suppressed by overexpression of UCP1. Meanwhile, ECM deposition-related markers Collagen I and Fibronectin were up-regulated under the stimulation of TGF-β1, which was also improved after UCP1 overexpression. Then, in order to explore whether CL316243 could play a comparable role in vitro, CL316243 was added to HK2 cells at the same time as TGF-β1 stimulation. The results showed that the ECM deposition and EMT level under TGF-β1 stimulation were both ameliorated after CL316243 treatment (Fig. 4G, H).

### Upregulation of UCP1 and CL316243 treatment improve renal interstitial fibrosis by inhibiting oxidative stress response

**Fig. 5:**
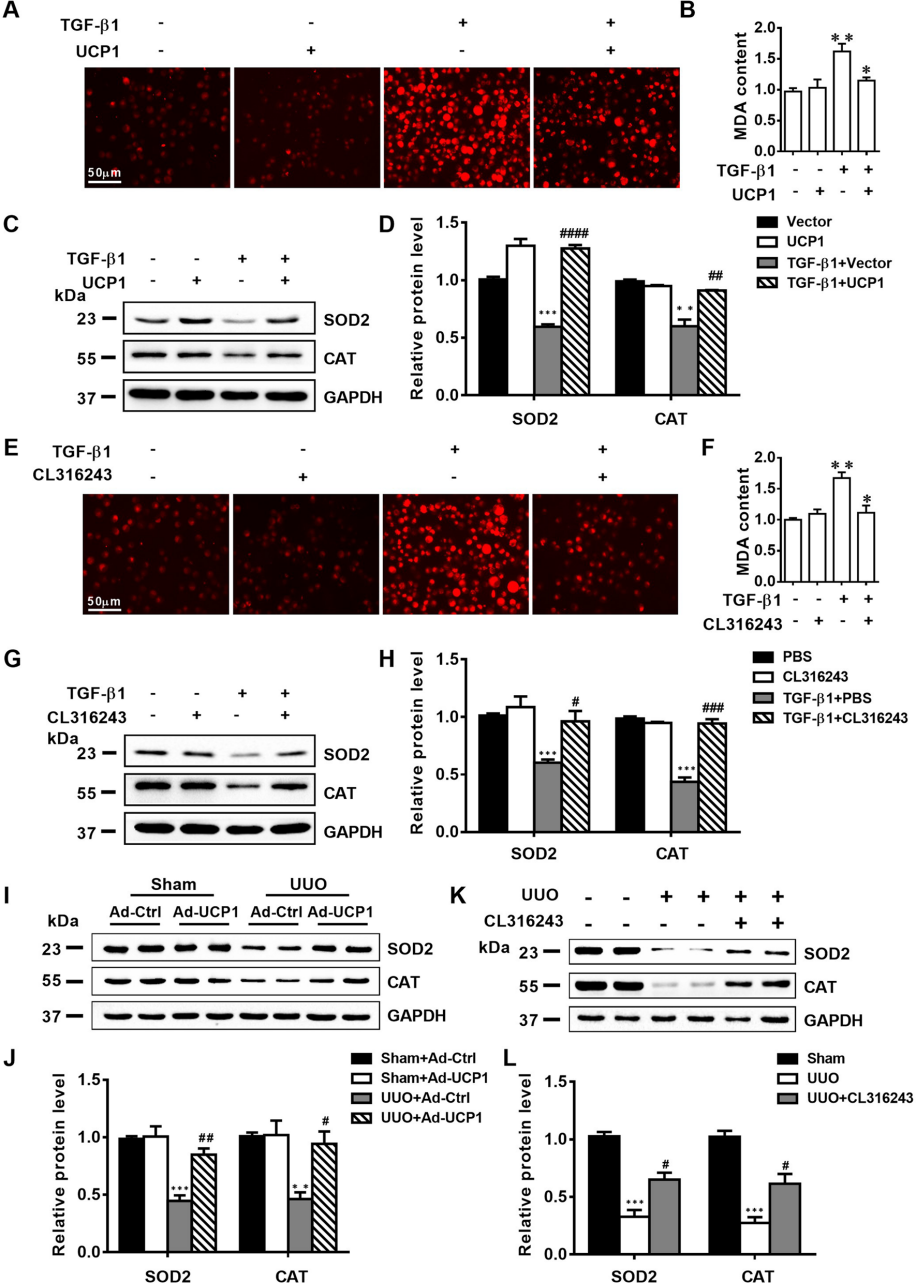
Upregulation of UCP1 and CL316243 treatment inhibits ROS production in UUO mice and TGF-β1 stimulated TECs. **A** Immunofluorescence images of ROS in HK2 cells in different cell groups. **B** Relative MDA content in HK2 cells specified in A. **C** Representative images and **D** corresponding quantification of SOD2 and CAT in HK2 cells specified in A. **E** Immunofluorescence images of ROS in HK2 cells in different cell groups. **F** Relative MDA content in HK2 cells specified in E. **G** Representative images and **H** corresponding quantification of SOD2 and CAT in HK2 cells defined in E. **I** Representative images and **J** corresponding quantification of SOD2 and CAT in Sham and UUO mice with control or UCP1-expressing adenovirus injection (n = 5). **K** Representative images and **L** corresponding quantification of SOD2 and CAT in Sham and UUO mice with or without CL316243 therapy (n = 5). * p < 0.05, ** p < 0.01, *** p < 0.001, **** p < 0.0001 vs. Ctrl or Sham; # vs. TGF-β1 or UUO

According to above research results, we have basically affirmed the important role of UCP1 in renal interstitial fibrosis, but its specific mechanism remains to be clarified. In our previous studies, we found that UCP1 affected lipid accumulation, apoptosis and autophagy in AKI [13], but in this model, UCP1 has not been found to have these functions (Additional file 3: Fig. S3). Oxidative stress is one of the crucial factors for the formation of renal interstitial fibrosis. During the occurrence and development of renal interstitial fibrosis, its continuous existence can stimulate multiple cytokines and activate a variety of signaling pathways [18, 19]. Studies have demonstrated that UCP1 decreased the oxidative phosphorylation efficiency and participated in the control of mitochondrial ROS production. Our study also found that through ROS fluorescence detection kit, the fluorescence intensity increased significantly in TGF-β1 stimulated HK2 cells, while decreased after overexpression of UCP1 (Fig. 5A). The content of MDA is usually used to indicate the degree of lipid peroxidation. Based on the results of MDA detection kit, UCP1 overexpression apparently decreased the MDA content in TGF-β1 stimulated HK2 cells (Fig. 5B). Meanwhile, the protein and mRNA level of antioxidant enzymes SOD2 and CAT, which could protect cells from oxygen free radicals, evaluated after overexpression of UCP1 (Fig. 5C, D, Additional file 2: Fig. S2C, D). Furthermore, we treated HK2 cells with CL316243 to explore whether it can inhibit the oxidative stress response induced by TGF-β1. From Fig. 5E–H, results displayed that after using CL316243, the fluorescence intensity of ROS provoked by TGF-β1 abated evidently, the content of MDA decreased, and the expression of antioxidant enzymes SOD2 and CAT restored, which were similar to the effect of UCP1 lentivirus transfection. Finally, we validated the impact of UCP1 on oxidative stress in vivo. The results of western blot analysis revealed that the expressions of SOD2 and CAT, which were decreased after UUO modeling, were significantly elevated in mice injected with adenovirus overexpressing UCP1 in renal parenchyma and treated with CL316243 (Fig. 5I–L).

### UCP1 alleviates ROS production by regulating the stability of SIRT3

**Fig. 6:**
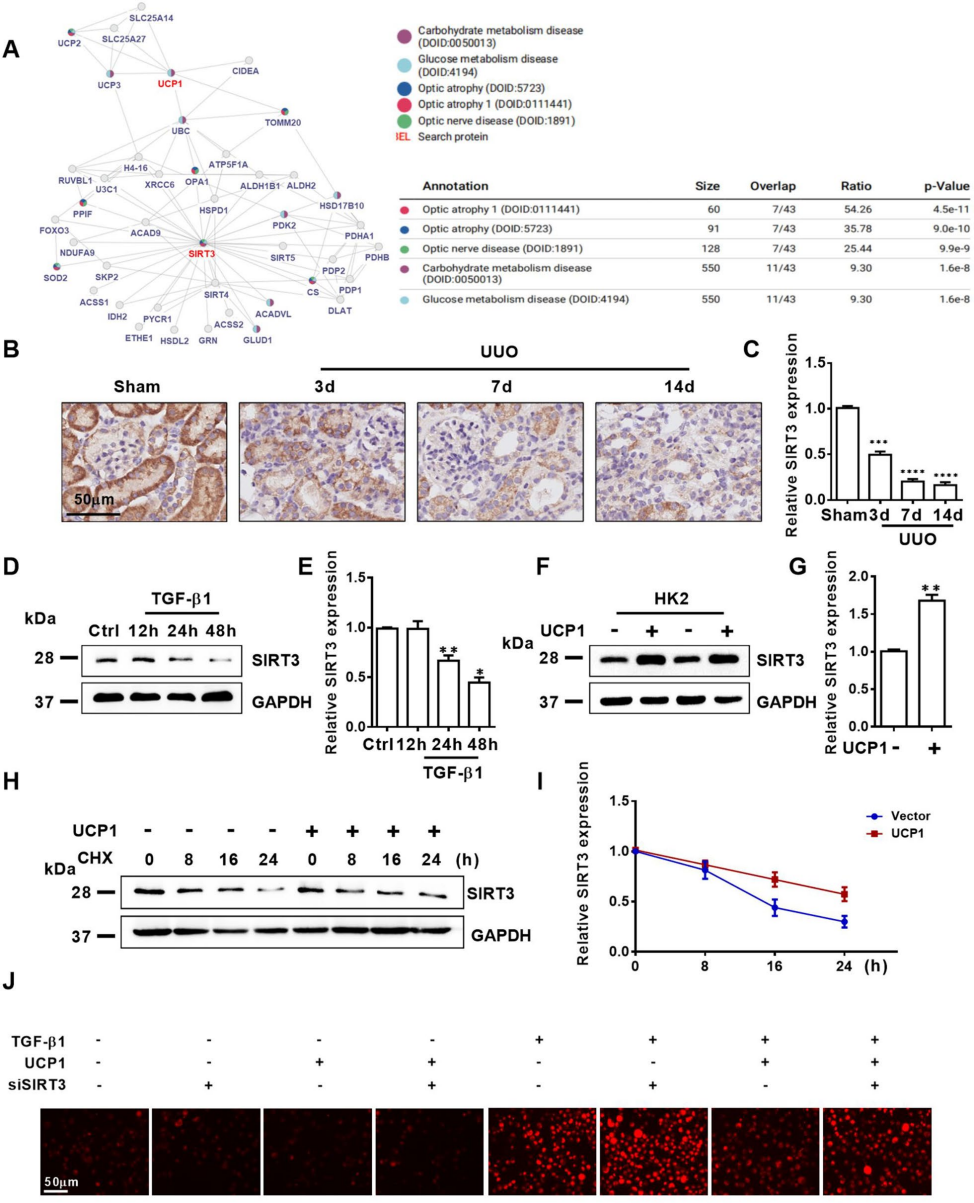
UCP1 alleviates ROS production in renal fibrosis by regulating the protein stability of SIRT3. **A** inBio Discover website showing highly correlation between UCP1 and SIRT3. **B** Photomicrographs displaying immunohistochemical staining against SIRT3 in UUO mice for 3, 7, and 14 days. **C** Aggregated statistics of integrated optical density (IOD)/area (n = 5). **D** Western blot results and **E** corresponding quantification of SIRT3 in HK2 cells exposed to TGF-β1 for 12, 24 and 48 h. **F** Western blot results and **G** corresponding quantification of SIRT3 in HK2 cells in different cell groups. **H** Western blot images and **I** corresponding quantifications of SIRT3 in different cell groups. **J** Immunofluorescence images of ROS in HK2 cells in different cell groups. * P < 0.05, ** p < 0.01, *** p < 0.001, **** p < 0.0001 vs. Ctrl or Sham

The above research results indicated that UCP1 mitigated the degree of EMT and abnormal accumulation of ECM by inhibiting oxidative stress response, thereby delaying renal interstitial fibrosis, however the specific mechanism of its regulation of oxidative stress response has not been elucidated. SIRT3 is one of the highly conserved sirtuin family members in evolution. It can improve ROS scavenging enzyme activity, stabilize mitochondrial function and deacetylate related acetylated proteins, thus inhibiting ROS accumulation in mitochondria. Since both UCP1 and SIRT3 were mainly located in mitochondria and had similar functions, and the prediction from the inBio Discover website found that UCP1 and SIRT3 might be strongly related (Fig. 6A), the relevance between them was further explored. Firstly, through immunohistochemical staining analysis, it was found that SIRT3 mainly existed in renal tubules, and the expression decreased gradually with the aggravation of interstitial fibrosis in UUO mice model (Fig. 6B, C). In vitro, western blot analysis displayed that the expression of SIRT3 reduced with the prolongation of TGF-β1 stimulated HK2 cells (Fig. 6D, E). SIRT3 was overexpressed by SIRT3-overexpressing lentivirus, and the results showed that SIRT3 could also improve TGF-β1-induced EMT, ECM aggregation and ROS production (Additional file 4: Fig. S4). Meanwhile, overexpression of UCP1 could significantly increase the expression of SIRT3 (Fig. 6F, G). To further investigate whether UCP1 affected the stability of SIRT3, control cells and UCP1 overexpressed cells were treated with the protein biosynthesis inhibitor cycloheximide (CHX) for a specified time. The results showed that SIRT3 protein levels in control cells decreased significantly over time after CHX treatment, whereas in cells overexpressing UCP1, SIRT3 protein levels decreased only mildly after CHX treatment (Fig. 6H, I). Overall, UCP1 enhanced SIRT3 protein stability by inhibiting protein degradation. Based on these findings, in order to verify the significance of SIRT3 in the regulation of renal fibrosis by UCP1, the expression of SIRT3 was knocked down through siRNA. The results showed that knocking down SIRT3 significantly reversed EMT-related marker α-SMA and E-cadherin, Fibronectin, antioxidant enzymes SOD2 and CAT, ROS reduction caused by UCP1 overexpression in TGF-β1 stimulated group (Fig. 6J, Additional file 5: Fig. S5).

**Fig. 7:**
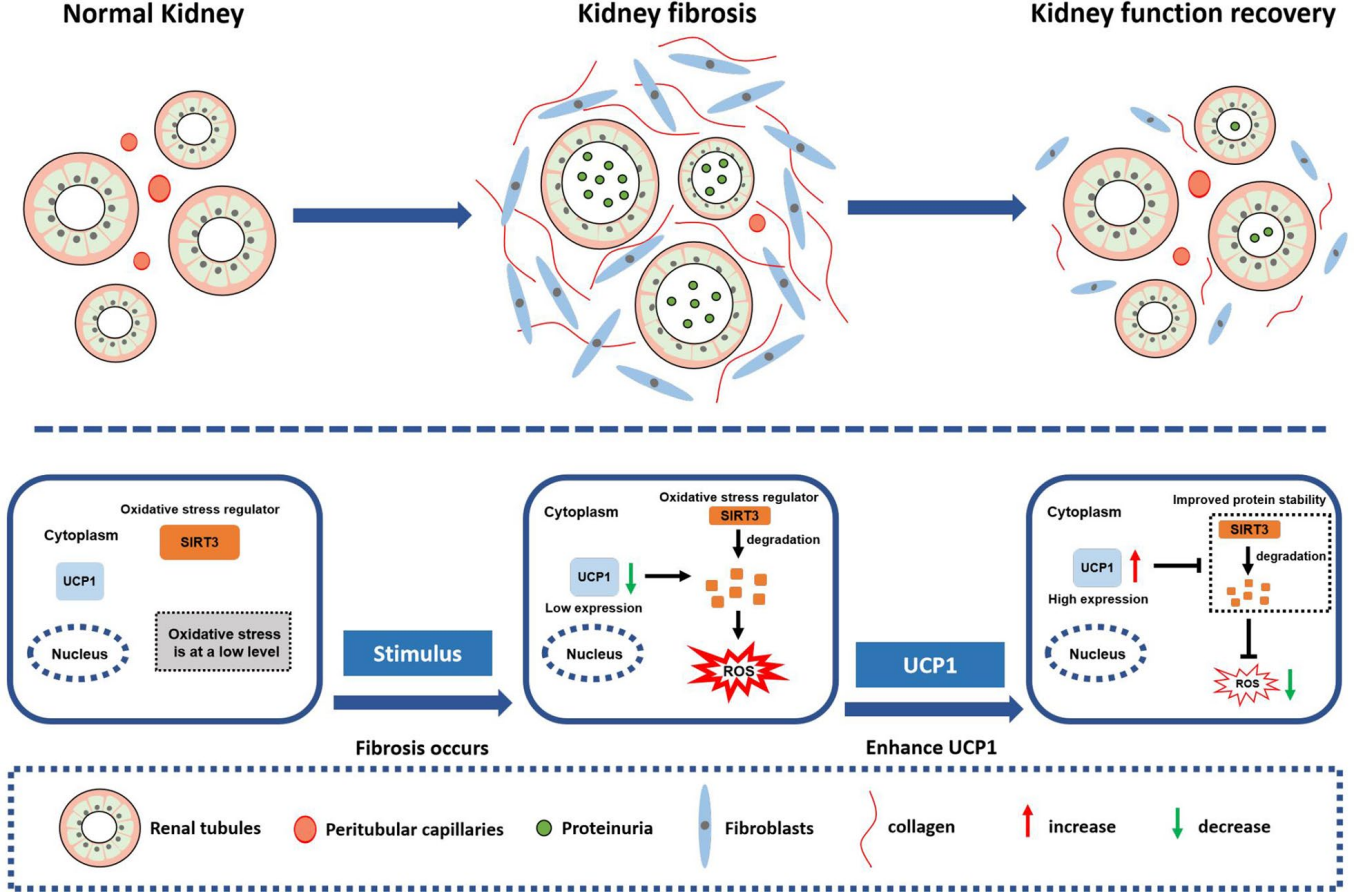
The proposed model illustrates the role and mechanism of UCP1 in renal interstitial fibrosis. The expression of UCP1 in renal tubules is relatively high under normal conditions, and oxidative stress is at a low level. When renal interstitial fibrosis occurs due to various etiologies, the expression of UCP1 decreases, resulting in a reduction in the protein stability of SIRT3 and subsequent degradation, leading to oxidative stress production. Elevating UCP1 expression can inhibit the occurrence of oxidative stress by stabilizing SIRT3, thereby reducing EMT and ECM accumulation, and ultimately alleviating renal interstitial fibrosis

In summary, UCP1 expression was notably downregulated in renal interstitial fibrosis. Elevating the expression of UCP1 inhibited the occurrence of oxidative stress by stabilizing SIRT3, thereby reducing EMT and ECM accumulation, and ultimately alleviating renal interstitial fibrosis (Fig. 7).

## Discussion

CKD is a chronic disease caused by a variety of reasons, mainly characterized by structural damage and dysfunction of the kidneys. Renal fibrosis is a common pathological alter from CKD to ESRD, which is mainly manifested in abnormal secretion of fibrin, excessive proliferation of glomerular mesangial cells, excessive accumulation of extracellular matrix, gradually forming renal tubulointerstitial fibrosis and glomerulosclerosis. However, specific diagnostic markers are lacking in the early stage. With the progress of fibrosis, renal parenchyma will be gradually destroyed with decline of renal function, and finally cause irreversible damage to the kidney. Once entering the ESRD, we can only take renal replacement therapy which requires high treatment costs and poor prognosis [20, 21]. Our research identified the new clinical biomarkers of CKD, and discovered the significance of UCP1/SIRT3/ROS pathway, which provides a new orientation for the research of CKD.

Uncoupling proteins (UCPs) are a class of carrier proteins that mediate mitochondrial proton H + transport and participate in oxidative phosphorylation uncoupling. Proton H + enters the mitochondrial matrix side through UCPs, the electrochemical potential energy is released in the form of heat, and ATP generation is reduced [22, 23]. Currently, six UCPs subfamily members have been found in mammals and UCP1 is specifically expressed in BAT [24]. However, in our previous studies, it was found that UCP1 was also present in the kidney, and was mainly expressed in renal tubules, while it was rarely expressed in renal medulla, renal papilla and glomerulus [13].

UCP1 is the first uncoupling protein found in the uncoupling protein family with oxidative phosphate uncoupling effect, and plays a vital role in thermogenic metabolism in vivo [25]. Previous studies have revealed that UCP1 mainly existed in BAT. UCP1 in mitochondria can directly convert the energy generated by the decomposition of glucose and fatty acids in food into heat energy instead of ATP. Studies have shown that UCP1 in BAT had a variety of functions, including mediating cold resistance, burning fat and glucose, preventing excessive fat accumulation in the body, and so on. It is the primary source of non-shivering heat production in mammals [26]. Recent studies have found that UCP1 was also expressed in mouse uterine smooth muscle cells and male reproductive and digestive tract smooth muscle cells [27]. For the past few years, studies have found that UCP1 plays an important role in AKI, and tubular injury in AKI is mainly manifested by increased apoptosis and activation of inflammation [13, 15]. However, in CKD, mitochondrial dysfunction, cell cycle arrest and cell senescence occur in renal tubules, as well as EMT, inducing persistent inflammation in renal fibrosis. At present, the role of UCP1 in CKD has not been studied. Our study found that UCP1 was decreased in patients and UUO animal model with renal fibrosis. Further research discovered that UCP1 overexpression reversed EMT and ECM accumulation both in vivo and in vitro. This proved that UCP1 was an important target of renal fibrosis, which played a key role in the occurrence and development in renal interstitial fibrosis.

CL316243 is a selective β3 receptor agonist and a potent stimulator of adipocyte lipolysis that increases thermogenesis and metabolic rate in BAT. CL316243 is currently widely used in obesity, diabetes and urinary incontinence related research. β3 receptor agonists belong to the family of G protein-coupled receptors, which activate intracellular adenylate cyclase (cAMP) through ligand binding, and in turn activates protein kinase A (PKA). cAMP-PKA signaling pathway activation can promote the transcription of UCP1 gene and increase UCP1 expression [28, 29]. Meanwhile, β3 receptor agonists also up-regulates the expression of UCP1 by activating the p38/MAPK bypass [30]. Studies have shown that CL316243 consumed lipids by promoting lipolysis of WAT and thermogenesis of BAT, and up regulated the expression of UCP1, playing a vital role in the remedy of obesity [17, 31]. In the present study, by intraperitoneal and tail vein injection of CL316243 in vivo, we found that the UCP1 expression was significantly elevated in the kidney, and after the intervention, the degree of renal interstitial fibrosis was alleviated. This indicated that CL316243 could act as an agonist of UCP1 in renal interstitial fibrosis, and might also be a potential treatment for CKD in the future.

The occurrence and development of renal interstitial fibrosis is complex, involving multiple pathogenic factors and mechanisms, such as inflammatory response, oxidative stress, and intrinsic renal cells apoptosis [4]. Oxidative stress refers to that when the body is exposed to harmful stimuli, the balance between the oxidative system and the antioxidant system in the body is broken, resulting in an abnormal increase in the production of ROS, which causes various damages. ROS is a class of small molecules with strong oxidative activity, which can directly attack biological macromolecules such as nucleic acids and proteins, eventually causing cell necrosis and apoptosis, tissue damage, inflammation and fibrosis [32]. Studies have revealed that the increase of ROS and the decrease of antioxidant enzyme activities were closely related to the occurrence and development of obstructive renal injury [33]. In the kidney, excess ROS directly induced pathological damage to various renal cells, stimulated the expression of fibrosis-related factors, promoted the fibroblasts proliferation and differentiation, reduced the number of mesangial cells and the degradation of ECM, and finally aggravated renal fibrosis progression [34, 35]. Nevertheless, UCP1-mediated uncoupling can scavenge oxygen free radicals generated during oxidative respiration and lessen the level of intracellular oxidative stress [14]. Therefore, we intervened by overexpression of UCP1 and CL316243 treatment in animal and cell models to detect the content of ROS and the expression of antioxidant enzymes. The results showed that the above intervention significantly reduced oxidative stress, which might be an vital mechanism of UCP1 in renal interstitial fibrosis.

SIRT3 is one of the members of the Sirtuins family, whose main function is to regulate the structure and function of mitochondria, participate in most oxidative stress processes, prevent the excessive production of ROS in mitochondria, and affect cell survival, proliferation, metabolism and aging and biological effects [36]. SIRT3 is abundant in human kidney, and studies have shown that SIRT3 could protect proximal tubular cells from palmitic acid-induced lipotoxicity by promoting mitochondrial oxidation and antioxidant capacity [37]. In this study, the expression of SIRT3 was significantly elevated after overexpression of UCP1, and the regulation of oxidative stress by UCP1 could be reversed by knocking down SIRT3, indicating that SIRT3 was the key intermediate molecule of UCP1 in regulating oxidative stress in renal interstitial fibrosis. It is worth noting that previous studies have confirmed that SIRT3, as a predictive target for NRF2, can protect against ER stress-induced damage [38], and NRF2 is also an important molecule in regulating oxidative stress [39]. Therefore, the role and mechanism of NRF2 in CKD deserve further study. We further explored the specific regulatory mechanism of UCP1 on SIRT3. Gene expression regulation is a complex process in response to changes in environmental conditions, which makes the gene expression process in the cell in an orderly state in time and space. Post-translational modifications (PTMs) are considerable ways of protein function regulation, which influence the structure and function of proteins by changing the properties of protein, such as electrification, hydrophilicity/hydrophobicity and spatial structure. They are closely related to many systemic diseases such as nervous system, endocrine system, cardiovascular system, urinary system and so on [40–42]. We used CHX, a protein biosynthesis inhibitor, to treat control and UCP1-overexpressing cells and the results showed that SIRT3 protein levels in control cells decreased significantly over time after CHX treatment, whereas in cells overexpressing UCP1 only slightly decreased, suggesting that UCP1 enhanced the protein stability of SIRT3 by restraining protein degradation.

## Conclusions

Overall, this is the systematic analysis of UCP1 differences in renal interstitial fibrosis and illustrates UCP1 as a prominent marker of the disease. Upregulation of UCP1 and CL316243 treatment can activate SIRT3 protein stability dependent oxidative stress pathway, inhibit EMT and ECM accumulation, and relieve the progression of renal interstitial fibrosis. This study identifies new targets for CKD treatment and opens up possibilities for the development of new drugs and combination therapies.

## Supplementary Information

**Additional file 1: Figure S1.** Urine indicators of UUO model and sham model. **A** Relative 24-hour urinary protein quality of UUO model and sham model. **B** Relative creatine ratio of UUO model and sham model. **C** Relative Kim-1 concentration in urine of UUO model and sham model. NS p > 0.05 vs. Sham.

**Additional file 2: Figure S2.** MRNA levels of EMT and oxidative stress related indicators after UCP1 overexpression. **A**, **B** Relative mRNA levels of E-cadherin and α-SMA in Sham and UUO mice with UCP1-expressing adenovirus or blank control injection. **C**, **D** Relative mRNA levels of SOD2 and CAT in Sham and UUO mice with UCP1-expressing adenovirus or blank control injection. * p < 0.05, ** p < 0.01, *** p < 0.001, **** p < 0.0001 vs. Sham; # vs. UUO.

**Additional file 3: Figure S3.** Effects of UCP1 overexpression on lipid deposition, apoptosis and autophagy. **A** Oil red staining of HK2 cells exposed to TGF-β1 with or without UCP1 overexpression. **B** TUNEL assay fluorescence images of HK2 cells exposed to TGF-β1 with or without UCP1 overexpression. **C** Western blot images and corresponding quantifications of LC3 in HK2 cells exposed to TGF-β1 with or without UCP1 overexpression. NS p > 0.05 vs. TGF-β1.

**Additional file 4: Figure S4.** Upregulation of SIRT3 inhibits ROS production, EMT and ECM accumulation. **A** Western blot images and **B** corresponding quantifications of Fibronectin, E-cadherin and α-SMA in HK2 cells in different cell groups. **C** Immunofluorescence images of ROS in HK2 cells in different cell groups. * p < 0.05, ** p < 0.01, *** p < 0.001, **** p < 0.0001 vs. Ctrl; # vs. TGF-β1.

**Additional file 5: Figure S5.** UCP1 alleviates renal fibrosis and ROS production by regulating SIRT3. **A** Western blot images and **B** corresponding quantifications of SOD2, CAT, Fibronectin, E-cadherin and α-SMA in HK2 cells in different cell groups. NS p > 0.05, * p < 0.05, ** p < 0.01, *** p < 0.001, **** p < 0.0001.

## Data Availability

All experimental datasets generated for this study are included in the article/Additional file.

## Abbreviations

UCP1: Uncoupling protein-1
CKD: Chronic kidney disease
BAT: Brown adipose tissue
ESRD: End-stage renal disease
UUO: Unilateral ureteral obstruction
EMT: Epithelial mesenchymal transformation
ROS: Reactive oxygen species
ECM: Extracellular matrix
HE: Hematoxylin
CHX: Cycloheximide
PKA: Protein kinase A
