# Supplementary figures and images for: UCP1 alleviates renal interstitial fibrosis progression through oxidative stress pathway mediated by SIRT3 protein stability
Source: J Transl Med. 2023 Aug 2;21:521. doi: 10.1186/s12967-023-04376-0 (PMC10399010; doi:10.1186/s12967-023-04376-0)

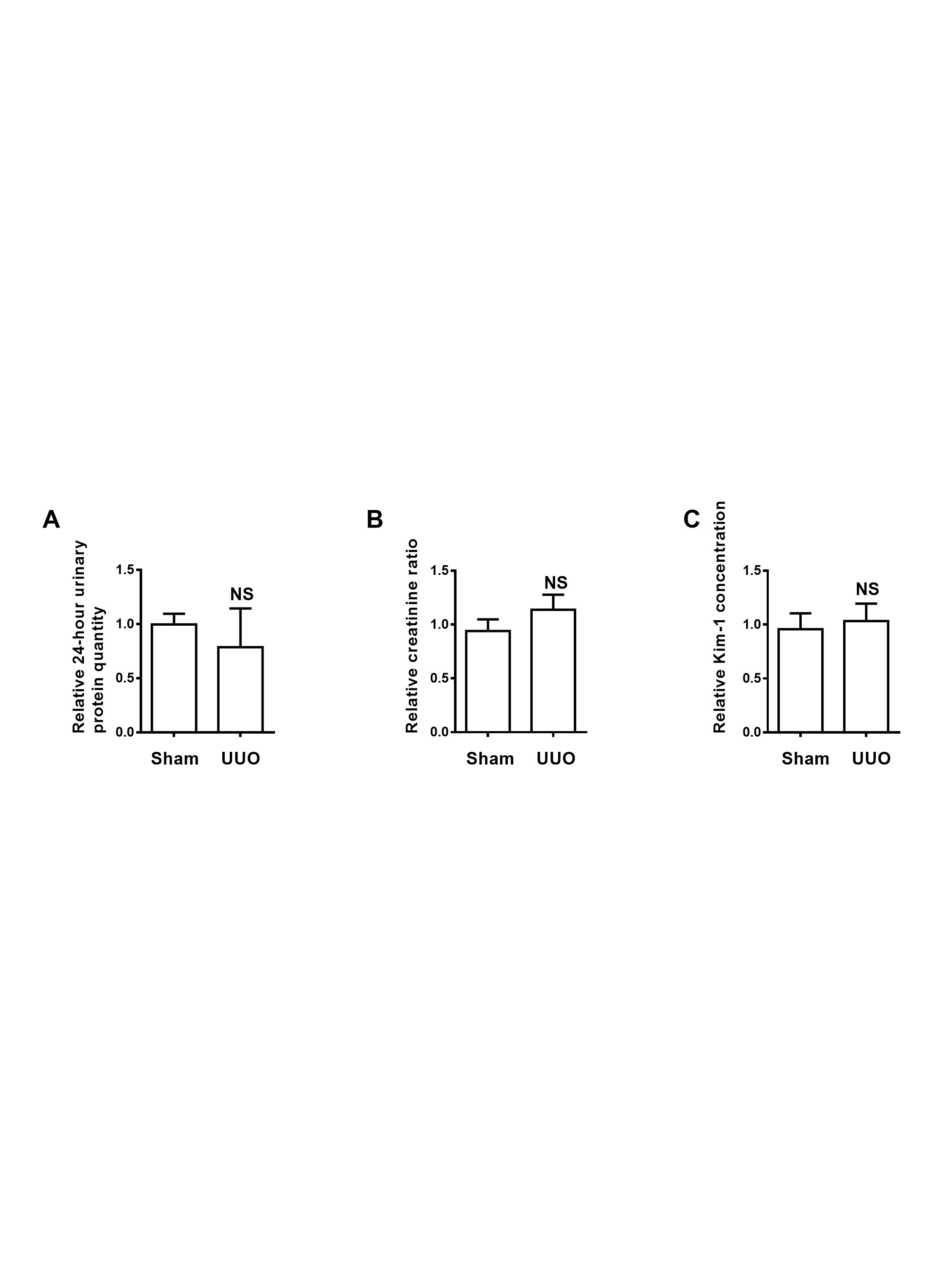

Supplement: Supplementary file 1 — Additional file 1: Figure S1. Urine indicators of UUO model and sham model. A Relative 24-hour urinary protein quality of UUO model and sham model. B Relative creatine ratio of UUO model and sham model. C Relative Kim-1 concentration in urine of UUO model and sham model. NS p > 0.05 vs. Sham. [file 12967_2023_4376_MOESM1_ESM.jpg]

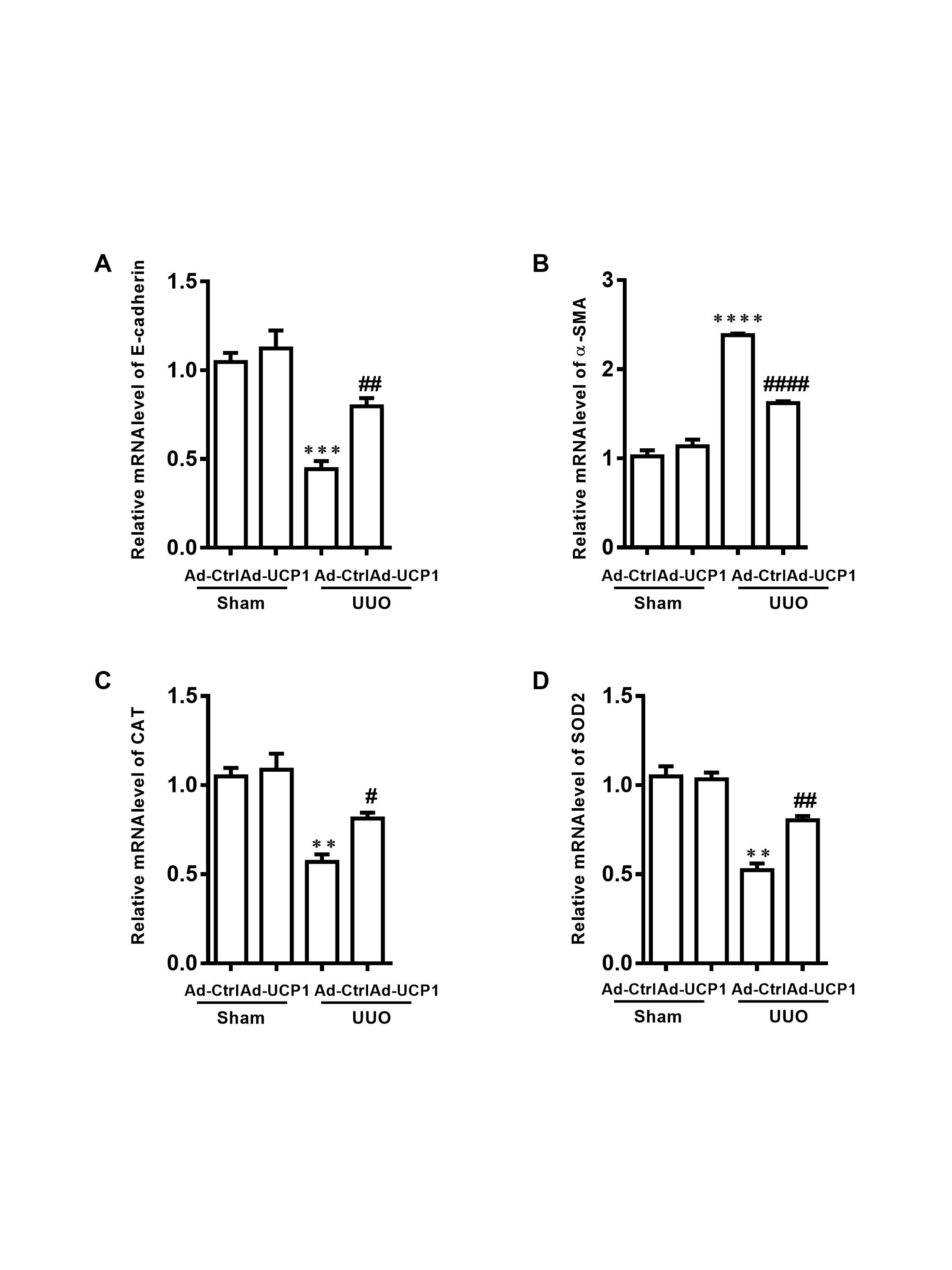

Supplement: Supplementary file 2 — Additional file 2: Figure S2. MRNA levels of EMT and oxidative stress related indicators after UCP1 overexpression. A, B Relative mRNA levels of E-cadherin and α-SMA in Sham and UUO mice with UCP1-expressing adenovirus or blank control injection. C, D Relative mRNA levels of SOD2 and CAT in Sham and UUO mice with UCP1-expressing adenovirus or blank control injection. * p < 0.05, ** p < 0.01, *** p < 0.001, **** p < 0.0001 vs. Sham; # vs. UUO. [file 12967_2023_4376_MOESM2_ESM.jpg]

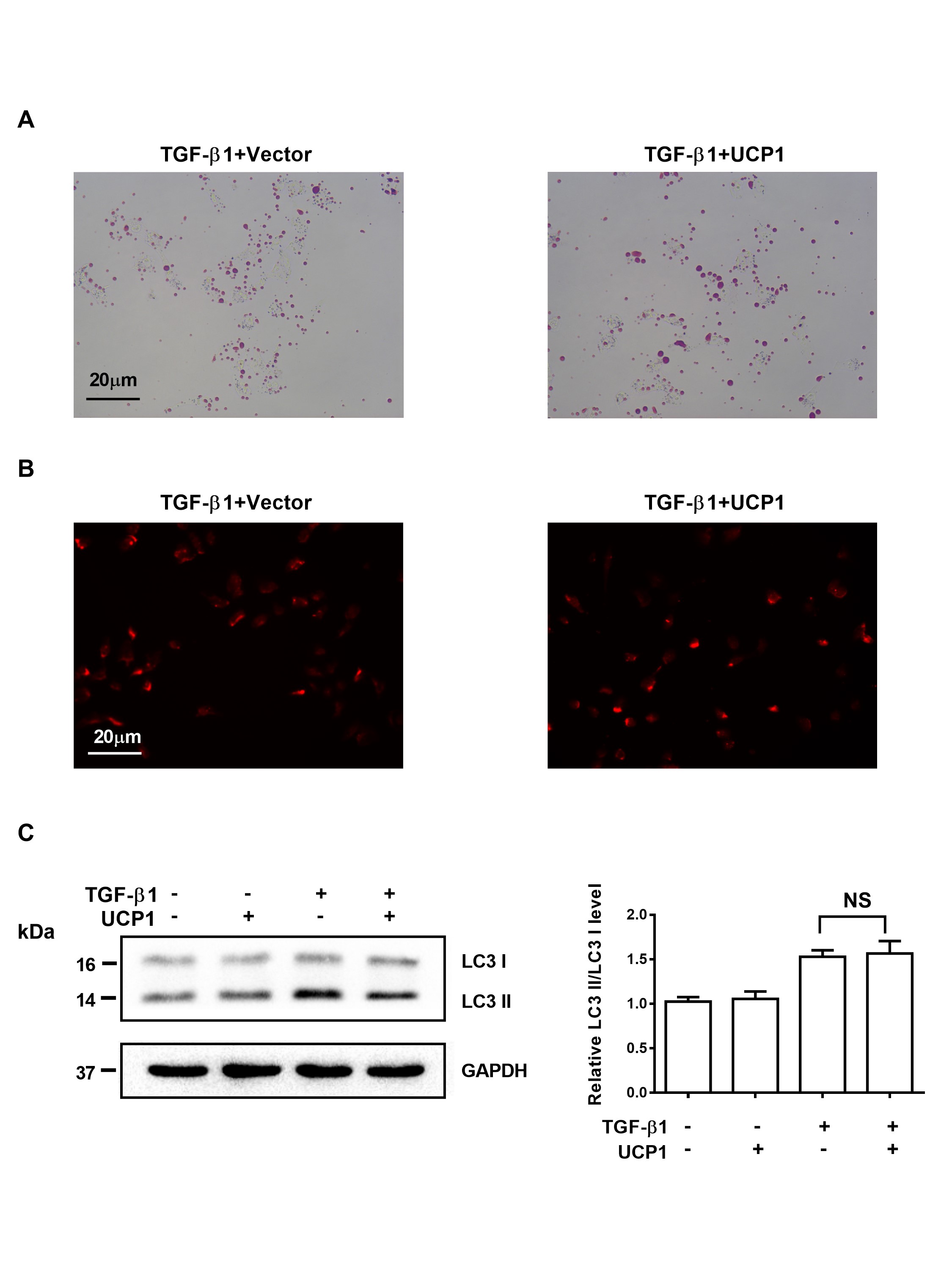

Supplement: Supplementary file 3 — Additional file 3: Figure S3. Effects of UCP1 overexpression on lipid deposition, apoptosis and autophagy. A Oil red staining of HK2 cells exposed to TGF-β1 with or without UCP1 overexpression. B TUNEL assay fluorescence images of HK2 cells exposed to TGF-β1 with or without UCP1 overexpression. C Western blot images and corresponding quantifications of LC3 in HK2 cells exposed to TGF-β1 with or without UCP1 overexpression. NS p > 0.05 vs. TGF-β1. [file 12967_2023_4376_MOESM3_ESM.jpg]

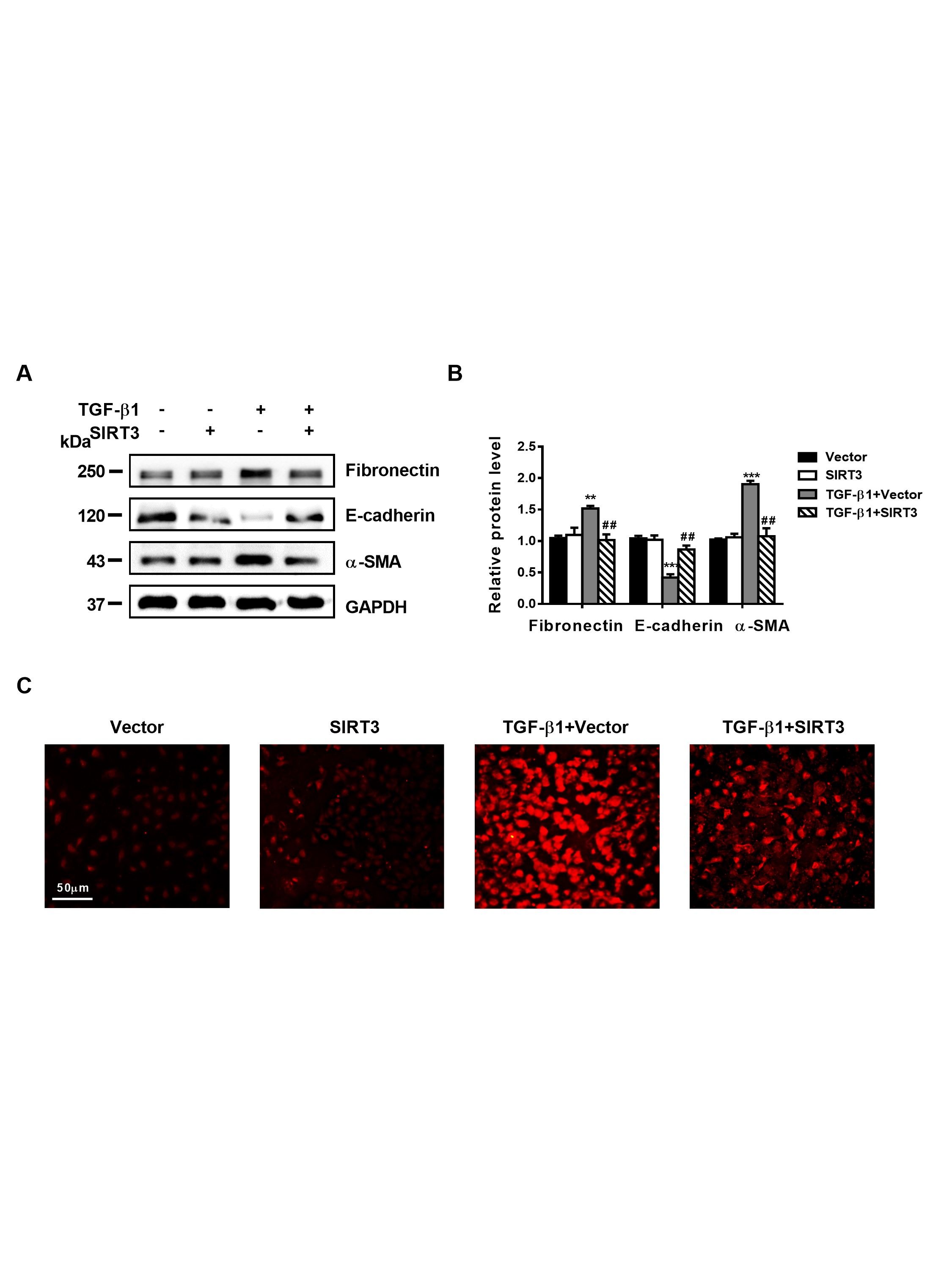

Supplement: Supplementary file 4 — Additional file 4: Figure S4. Upregulation of SIRT3 inhibits ROS production, EMT and ECM accumulation. A Western blot images and B corresponding quantifications of Fibronectin, E-cadherin and α-SMA in HK2 cells in different cell groups. C Immunofluorescence images of ROS in HK2 cells in different cell groups. * p < 0.05, ** p < 0.01, *** p < 0.001, **** p < 0.0001 vs. Ctrl; # vs. TGF-β1. [file 12967_2023_4376_MOESM4_ESM.jpg]

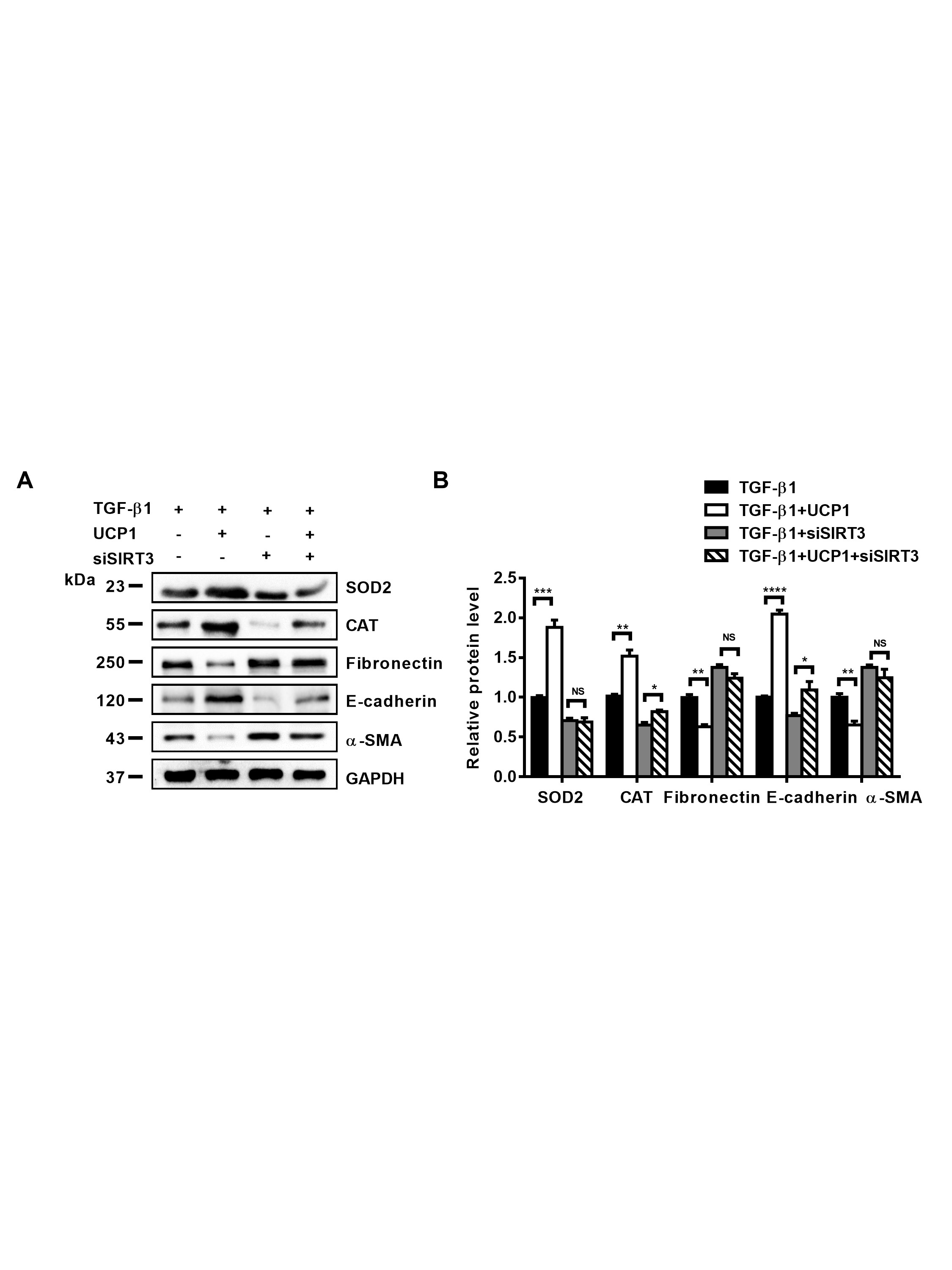

Supplement: Supplementary file 5 — Additional file 5: Figure S5. UCP1 alleviates renal fibrosis and ROS production by regulating SIRT3. A Western blot images and B corresponding quantifications of SOD2, CAT, Fibronectin, E-cadherin and α-SMA in HK2 cells in different cell groups. NS p > 0.05, * p < 0.05, ** p < 0.01, *** p < 0.001, **** p < 0.0001. [file 12967_2023_4376_MOESM5_ESM.jpg]
